# What constitutes ‘good’ home care for people with dementia? An investigation of the views of home care service recipients and providers

**DOI:** 10.1186/s12877-021-02727-4

**Published:** 2022-01-11

**Authors:** Anita M. Y. Goh, Meg Polacsek, Sue Malta, Colleen Doyle, Brendan Hallam, Luke Gahan, Lee Fay Low, Claudia Cooper, Gill Livingston, Anita Panayiotou, Samantha M. Loi, Maho Omori, Steven Savvas, Jason Burton, David Ames, Samuel C. Scherer, Nadia Chau, Stefanie Roberts, Margaret Winbolt, Frances Batchelor, Briony Dow

**Affiliations:** 1 National Ageing Research Institute, Parkville, VIC Australia; 2 The University of Melbourne, Parkville, VIC Australia; 3 Melbourne Neuropsychiatry Centre, Parkville, VIC Australia; 4 Royal Melbourne Hospital, PO Box 2127, Melbourne, VIC 3050 Australia; 5 Benetas, Melbourne, VIC Australia; 6 Institute of Epidemiology & Health Care, University College London, London, UK; 7 LaTrobe University, Melbourne, VIC Australia; 8 University of Sydney, Sydney, NSW Australia; 9 Division of Psychiatry, University College London, London, United Kingdom; 10 Safer Care, Melbourne, VIC Australia; 11 Monash University, Clayton, VIC Australia; 12 dementia360, Perth, Western Australia Australia; 13 Academic Unit for Psychiatry of Old Age, Kew, VIC Australia

**Keywords:** Caregivers, In-home care, Codesign, Stakeholder, Dementia

## Abstract

**Background:**

Our objective was to explore what people receiving and providing care consider to be ‘good’ in-home care for people living with dementia.

**Methods:**

We conducted 36 in-depth interviews and two focus groups with key stakeholders in Australia in the first quarter of 2018. Participants included those receiving care (4 people living with dementia, 15 family carers) or providing care (9 case managers, 5 service managers, 10 home care workers). Qualitative thematic analysis was guided by Braun and Clarke’s six-step approach.

**Results:**

Consensus was reached across all groups on five themes considered as important for good in-home dementia care: 1) Home care workers’ understanding of dementia and its impact; 2) Home care workers’ demonstrating person-centred care and empathy in their care relationship with their client; 3) Good relationships and communication between care worker, person with dementia and family carers; 4) Home care workers’ knowing positive practical strategies for changed behaviours; 5) Effective workplace policies and workforce culture. The results contributed to the co-design of a dementia specific training program for home care workers.

**Conclusions:**

It is crucial to consider the views and opinions of each stakeholder group involved in providing/receiving dementia care from home care workers, to inform workforce training, education program design and service design. Results can be used to inform and empower home care providers, policy, and related decision makers to guide the delivery of improved home care services.

**Trial registration:**

ACTRN 12619000251123.

## Background

People with dementia often want to continue to live at home rather than move to residential care [1], and there are physical, emotional, cognitive, social, and economic benefits to older people staying at home if they can, including preserving physical and mental health [2], independence and autonomy [3],and maintaining connections to family, friends, social groups, neighbourhoods, and communities [4]. Although family carers provide much of the care, paid in-home care is often needed as an adjunct to maintain independence, health, and safety of the person with dementia, particularly as symptoms progress [5–7].

Paid in-home care workers are typically employed by a service provider company and provide a range of services from personal care, domestic tasks, transport, and home maintenance. Home care workers also play a role in their clients’ psychological, intellectual, emotional and social needs [8, 9], and enable people with dementia to remain socially connected within their community [10]. Finally, they support family carers, reducing their stress and providing them respite time [10]. Randomized controlled trials have shown home care contributes significantly to reduced hospital admission, delayed institutionalization, and improved quality of life [11–13].

In Australia, older people receive government-subsidised home care via the Commonwealth Home Support Programme (CHSP) or the Home Care Package program. Home Care Packages are delivered by government-approved service providers and provide higher levels of support than CHSP and are funded at a higher level. Packages are means tested with annual and lifetime caps to co-contributions, and there are dementia and cognition supplements for those with moderate to severe cognitive impairment [14]. Although expensive, people can also elect to pay the full cost via private providers who don’t receive government funding (for example if they are found to be ineligible for Australian Government-subsidised aged care services, or are found to be eligible but are waiting for funded services to become available). The majority of home care service providers provide generic care for a wide range of needs, rather than being dementia-specific.

In terms of dementia training, nationally accredited dementia training is available (including from government-funded bodies such as Dementia Australia and Dementia Training Australia), but it is not currently mandatory to complete dementia training to work as a home care worker. Many home care workers may have undertaken short-course vocational training, but dementia content varies greatly and is typically very limited [15]. For example, most workers complete a Certificate III in Individual Support (previously known as Certificate III in Aged care) which does not include any compulsory dementia content. Therefore, many home care workers have very limited dementia specialist training and knowledge [16]. The 2021 Australian Royal Commission into Aged Care Quality and Safety (the highest level of Government inquiry) identified the urgent and critical need for specialist dementia care training for aged care workers, including home care workers [17].

### What does the existing literature tell us about the quality of home care?

Although many people with dementia want to “age in place”, it is unclear what constitutes good or quality in-home care, in terms of which care models are the most effective, equitable, and accessible. The concept of quality in aged care is complex and difficult to define. It may include efficiency, effectiveness, safety, comfort, dignity, service accessibility, staff attitudes and behaviour, continuity and reliability of staff, clinical care, physical environment and choice [18]. Ratcliffe et al. [19] in a comprehensive review of literature pertaining to quality of care and/or person-centred care in aged care within the last decade, noted that most of the research in this area has focused on residential aged care, and not on community services. They found that the general public values an aged care system that ensures older people feel safe and comfortable, and are treated with dignity and respect. The general public also values an aged care system that provides services and support for health and well-being, and has a workforce with appropriate skills and training. Dyer et al. [20] concluded that most innovative models of dementia care (in Australia, and internationally) have little evidence of their effectiveness at improving care recipients’ outcomes, and still need rigorous evaluation before they can be implemented and scaled up for service delivery in the community.

Focusing on home care, Lord et al. [21] built an evidence-informed model to best support people with dementia to live independently at home. Their “NIDUS theoretical model of independence at home” includes ten values and approaches to best support people living with dementia at home. These are: (1) Care should be person-centred, compassionate, and include the carer and other important relationships; (2) Care decisions and strategies should balance their right to protection and autonomy (which may conflict), and calculated risks may be necessary in order to have more freedom and independence; (3) Value and continue their connections with previous roles and social networks, as these are part of someone’s identity; (4) As much as possible, modify their home and environment to be dementia-friendly; (5) Use tailored activities for each person; (6) Identify and prioritise the needs and goals of the person with dementia, and their carers; (7) If possible, use strategies to reduce disability; (8) Enable self-management; (9) Provide a single contact; and (10) Continuity of care.

Although the model by Lord and colleagues was published in 2019, there has been limited implementation of this model, and currently, there is no standard and widely accepted model of care for in-home care for people with dementia, nor a definition of “good care” or “quality care” across the sector to inform these models of care. It is also unclear what outcomes would or should constitute “good quality care” and how to measure these (for example, outcomes could include quality of life, health outcomes, transition to residential care, and/or profit margins) [ 21]. Even less is known about what constitutes ‘good’ home care from the perspective of those receiving and providing care [16]. The lack of a clear and fundamental understanding of what constitutes good care, particularly by those who give *and* receive care, affects our ability to design effective models of care, and to understand and measure how ‘effective’ the care is.

In order to fill this gap in basic understanding of what constitutes ‘good home care’, this study aimed to describe the views of key stakeholders (people with dementia, family carers, home care workers, case managers and service managers) on what they considered important for high-quality in-home dementia care. In this study, we were interested in what the term “good care” meant to the people giving and receiving home care services, rather than eliciting their opinions of any predefined definitions, structures, or formal indicators of quality care (for example, the National Aged Care Mandatory Quality Indicators). This project was part of the larger Promoting Independence Through quality dementia Care at Home (PITCH) project, where participants co-designed an evidence-based dementia training program for home care workers, which is being evaluated in a randomised controlled trial (ACTRN 12619000251123).

## Methods

### Research design

A qualitative approach to data collection and analysis was used, with the consolidated criteria for reporting qualitative studies (COREQ) 32-item checklist as a reporting framework [22]**.**

### Participant recruitment

Participants were people with dementia, family carers, and people who provide paid home care for people with dementia. Using purposive sampling we recruited from eight service provider partners, who disseminated advertisements and letters of invitation to their staff and clients. Inclusion criteria for participants were: people of any age who: (i) had a diagnosis of dementia of any type and stage, who were receiving in-home care services funded by the Australian government; or (ii) family carers who care or previously cared for a person with a diagnosis of dementia, who met criteria (i). Individuals living with dementia were eligible to participate on their own or with their family carer.

Service provider participants were (iii) home care workers or (iv) service managers/case managers (including from non-profit, private sector, and providers focused on multicultural communities currently) providing in-home care services to people with dementia, funded by the Australian government. These participants provided care to a wide range of clients/service recipients, and were not dementia-specific service providers.

### Ethical considerations

Ethical approval was obtained from Austin Health Human Research Ethics Committee (HREC/17/Austin/537). Written informed consent was obtained from all participants, including from all four participants living with dementia, who were deemed to have capacity to do so. Two family carers (of the four participants with dementia) also co-signed proxy informed consent for their care recipient with dementia. The research staff were trained by a neuropsychologist to assess capacity to provide consent, determined by whether the potential participant: understood the nature of the research and their participation; appreciated the consequences of their participation; showed ability to consider alternatives including the option to not participate; and showed ability to make a reasoned choice. Written and verbal techniques were used to communicate, and the potential participant was asked to explain the details back to the researcher. Pseudonyms are used in this paper, except for two participants who wished to have their names used.

### Data collection

As per previous reports on this study [10], the PITCH study was undertaken in Melbourne in the state of Victoria, Australia. We used individual semi-structured qualitative interviews and focus groups to gather information about participants’ experiences of receiving or providing home care, how their experiences could be improved, and their opinions on what they considered important for high-quality home-based dementia care. These were conducted by AG, SM, LG, and BH in 2018. For people with dementia and family carers, interviews occurred at their home, and for service providers, at their place of work. No participants required an interpreter. There was no one else present during the interviews/focus groups besides the participants and researchers. Breaks were scheduled, and participants were also told (verbally and in writing via the consent form) that they could request a break at any time.

The interview questions were developed and piloted by a project advisory group, which included people with dementia, family carers, home care professionals, researchers, advocates, policymakers, and clinicians, ensuring diversity of views. The question schedule was flexible, started with open-ended questions and then more specificquestions. Although participants were invited to discuss any topics that were important to them in relation to dementia care, the focus was their experiences of receiving or giving in-home care for people with dementia (see Table 1).

**Table 1:** Sample of questions and prompts for all participants

Can you tell me about your experience of receiving/providing formal/paid home care?	
*(prompt)* Type and frequency of support	
*(prompt)* Alignment with current needs	
What is your opinion about the home care you are receiving/providing?	
Can you tell me what you like/do not like about the home care you receive/provide?	
• Type and frequency	
• Level of interaction/engagement	
• Appropriateness to current needs	
How would you describe the relationship between yourself and the other people involved in receiving/providing home care? Could it be improved?	
Can you tell me about the role the home care worker/client plays in your life?	
• Working as a team	
• Communication and engagement	
What do you think makes a great care worker, rather than a satisfactory (or just okay) one?	
Have you any suggestions for improvements that can be made to the kind of care you receive/provide from your current home care services program?	
How could your experience be improved?	
How do you think the care recipient’s experience could be improved?	
What do you think should be included in the PITCH training program?	
• Dementia knowledge	
• Practical strategies for providing care	
• Attitude and communication	

Field notes were not made during the interview or focus groups but occurred afterwards, and reported contextual details and non-verbal expressions for data analysis and interpretation. Each interview/focus group lasted approximately 90 min and was audio-recorded, then professionally transcribed verbatim and anonymised.

### Data analysis

Thematic analysis followed Braun and Clarke’s six-step approach [23]. Step 1 was familiarisation with the data, where transcripts were read several times to gain a comprehensive understanding of the participants responses. Initial data analysis was undertaken by SM, LG, MO, and MP, followed by independent review by AG where transcripts were read independently, and the coding framework reviewed. Themes were coded by hand between each interview conducted, in line with constructivist grounded approaches [24], then coded and clustered into themes in NVivo (Version 11) (Step 2 and 3). In Step 4 and 5 the researchers reviewed and defined the themes, asking reflexive and generative questions of the data, making comparisons and identifying and refining possible themes and sub-themes [25], including addressing preconceived notions to reduce bias or subjectivity [26]. Any differences in coding or thematic analysis between research team members was resolved through discussion until consensus was achieved. Step 6 is reporting the results via this publication.

#### Trustworthiness

We used the checklist for trustworthiness [27], and our study aligns with the Lincoln and Guba [28] criteria for trustworthiness (credibility, transferability, dependability, confirmability, and authenticity). Researchers summarised and verified participants’ responses after discussing a particular topic during the interview to increase the credibility of the study [26]. We also used the method of Synthesized Member Checking, which addresses the co-constructed nature of knowledge by providing participants with the opportunity to engage with, and add to, interview and interpreted data after their semi-structured interview [29]. This included all participants being invited to participate in group-based co-design workshops [30], which allowed them to see the outputs of their interviews and how their advice was used to inform the co-designed training program. We frequently communicated with participants regarding the training program, including invitations to attend multiple pilot testings of the training program. Thus, participants had multiple opportunities to verify their experiences or question the combined preliminary findings from the interviews. To enhance confirmability and dependability, independent review was used, where an additional expert researcher read transcripts independently, reviewed the coding framework, and peer-checked the codes extracted. The use of exemplars in this manuscript to illustrate each theme in the reporting supports authenticity of the findings and transferability to other situations. Transferability is also addressed in the detailed descriptions of the participants and the context of the study.

## Results

### Description of the sample

We conducted 36 individual interviews and two focus groups with home care staff (with three and four participants respectively). Four people living with dementia, 15 family carers, 10 home care workers, and 14 managers (9 case managers, 5 service managers) participated. All four people with dementia had received a diagnosis of later onset Alzheimer’s disease, and were at least 7 years post diagnosis. Demographic details are presented in Table 2. Paid and unpaid carers were predominantly women, with 100% of family carers and home care workers and 93% of managers being women.

**Table 2:** Demographic characteristics of focus group and interview participants (*n* = 43)

	People with dementia (n = 4)	Family carers (*n* = 15)	Home care workers (*n* = 10)	Case and Service Managers (*n* = 14)
Age in years (Mean, SD, range)	M = 80.75 SD = 11.50 Range = 70–95	M = 64.27 SD = 12.26 Range = 48–92	M = 52.11 SD = 7.34 Range = 43–62	M = 49.31 SD = 12.04 Range = 25–70
Gender	25% Women (*n* = 1) 75% Men (*n* = 3)	100% Women 0% Men	100% Women 0% Men	92.86% Women (*n* = 13) 7.14% Men (n = 1)
First language is English	100%	100%	70% *N* = 7 (other languages were Italian, Korean, Spanish)	79% *N* = 11 (other languages were Cantonese, Tagalog, Greek)
Mean age in years at diagnosis	M = 70.5 years SD = 7.07 Range = 66–75)			
Mean time in years since receiving diagnosis (range)	M = 8.5 years SD = 2.12 Range = 7–10			
Relationship to person with dementia		46.67% Spouse (n = 7) 53.33% Daughter (*n* = 8)		
Mean length of time in caring role in years		M = 6.1 years SD = 3.04 Range = 1–13)		

### Themes

Across all four stakeholder groups, five main themes were identified that were considered important to good in-home dementia care provided by home care workers. As there were no differences in the themes identified across all four groups, the findings are presented here by theme rather than by group membership:

1. Home care workers need more understanding of dementia and its impact
2. Home care workers should demonstrate person-centred care and empathy in their care
3. Good relationships and communication between care worker, person with dementia and family carers is important
4. Home care workers need to know practical strategies for changed behaviors
5. Effective workplace policies and workforce culture are important

### Theme 1. Home care workers need more understanding of dementia and its impact

There was a consensus across groups that home care workers needed to have a good understanding of dementia to provide good care and that there was currently a lack of appropriate training, understanding and knowledge about different types of symptoms and impacts (i.e. on person, behaviour response and health changes) of dementia. For example:

“*Homecare workers have to do a lot in their day so they come to you, who has a diagnosis of dementia, and then after you they might be looking after someone with autism or something so very different. They need more knowledge and plenty of information”* (Trevor, person living with dementia).

“*A bit of education. You know, at least the very basics of dementia … there’s this and this and this type and therefore you’ll see this and this and this behaviour. [Having dementia] doesn’t mean that they’re dumb”* (Rita, family carer).

Participants stated home care workers needed to understand that: 1) each individual’s response to their dementia journey presents differently, even if they have the same diagnosis; and 2) the progressive nature of dementia means an individual will change and have greater support needs over time. Home care workers described the need for more education, for example:

*[We need to] learn the different types [of dementia] and body language, especially for people with more advanced dementia … how to read, understand their body language, their facial expressions, their hands*” (Louise, home care worker).

Participants, specifically those receiving care, mentioned it is important for home care workers to know that some dementia symptoms are manifestations of a physical illness affecting the brain and do not reflect who that person really is, and then to apply this knowledge to individuals. A family carer stated:

“*I don’t think you need to know what a dendrite is … but understand the parts of the brain that are likely damaged, and the reason that somebody doesn’t have the ability to reason …. It stops you endlessly arguing over something that you can’t argue with, it’s fruitless*” (Ruth, family carer).

Managers also acknowledged the current training for home care workers was insufficient for quality dementia care. Exemplars included:

“*They come out [of their training] knowing about dementia and that it exists in the community, and they might have a relative with some form of dementia, but that’s about it. It’s not a well understood issue”* (Glenda, service manager).

### Theme 2. Home care workers should demonstrate person-centred care and empathy in their relationship with their client

All stakeholder groups agreed that having empathy as well as in-depth knowledge of the person with dementia – two commonly linked ideas – were essential to good, individually responsive, care. Participants said “knowing the person” was integral to their role because each person living with dementia was different, with unique personalities, varying interests, and diverse backgrounds. It was important home care workers acknowledged the personhood of the individual with dementia, regardless of their diagnosis.

“*The main element should be get[ting] to know your client … what their interests are, from start to finish … who they are. That’s the key to the good relationship, and a good working environment,*” (Leslie, home care worker)

The clients also highlighted this theme, stating:

*“(A good homecare worker)… Gets into the needs of the people that are there”* (Trevor, person living with dementia)

“*I think (home care workers) ought to speak to people before [they] start doing things. And give them a choice of feeling self-esteem. You don’t know one of those people might be really brilliant at something and [home care workers] never find out until they’re given a chance*” (Debra, person living with dementia).

A personalised approach was associated with higher quality care, as it recognised and supported a person’s individual needs and preferences, and their broader family context. Family carers said:

“*I guess knowing about dementia and knowing about the person you’re caring for are two different things. They probably need to have a little briefing on the person that they’re caring for...every person is different, aren’t they?”* (Helen, family carer)

“*You don’t walk into someone’s place and just making assumptions about dementia … it’s about person-centred care, personhood and respect*” (Marie, family carer).

The service provider managers agreed:

“*You can train them to be knowledgeable, but not empathetic. I think that’s either you’ve got it and you build on it, or you haven’t got it. But certainly exposure to people with dementia in all its forms couldn’t help but soften even a very hard heart*” (Glenda, service manager).

*“You earn their trust [the person with dementia]. You need to know about their past history, what they enjoyed. They’re the things that you discuss with the family. Do they like music? Do they want to go for a walk? Do they like knitting?”* (Milly, case manager).

### Theme 3. Effective relationships and communication between home carers, people living with dementia and family carers is important

There was consensus that forming a relationship between all parties who are directly involved in caring was important to providing good care. Each group mentioned home care workers provided good care when they behaved more like a friend (or even family member), focusing on the person and their preferences, rather than being task-focused. For example:

*“When anybody comes into the house I think we always end up laughing. And when they come back, we’re always on first names. ..They ended up as part of the family, as far as I’m concerned … they just became friends doing a job. I hated somebody who comes in as a ‘worker’ doing a job”* (Petro, person with dementia).

*“I could see that this man really just needs to have someone to chat to. So he would say, “Would you like a cup of tea?” and I’d say, “I’d love one, that’d be great.” So we would sit down and chat for an hour... – I’m glad this is anonymous – I know that his regular carer, because he told me, cleans and leaves. So she’s not actually staying there the whole amount of time... that’s someone who doesn’t appreciate the value of just being with someone for that time.”* (Grace, home care worker)

*“How to communicate with other people is very important for carers…Most of the people who come are lovely and fantastic … it’s a bit like having a friend, it’s not just ticking the boxes of ‘he’s had a shower’ .. It’s got to do with the quality of that time. I need to feel okay that they’re okay with [husband] … it’s as much engaging with me as it is with him*” (Carol, family carer).

Effective communication built rapport, trust, and respect, and optimised relationships and care, particularly when the family carers were also involved. Communication styles considered conducive to good care included respectful, compassionate, and person-centred styles; being able to use humour as a tool; and having the ability to communicate effectively to de-escalate situations. Family carers framed it as a “collaborative” effort:

*“I guess when they understand about doing ‘with’ and not ‘for’, or ‘to’.”* (Marie, family carer)

*“They have to listen to the other carer, because I'm the one that's doing it 24-hours a day, seven days a week. I'm living it.”* (Caroline, family carer).

Home care workers agreed that communication and liaison was key, for example:

*I had contact with the family and there was a communication book, so I could write what I had been doing with the client that day, and her family would also write comments. So that felt really good, because there was some liaison* (Grace, home care worker).

The need for effective communication in the direct care relationship was also recognised by managers, who stated that part of good home care was allowing family carers to share their experiences of caring for the person with dementia. Conversely, poor communication and rapport between home care workers and family carers caused feelings of disempowerment and paternalism, hindering quality care.

*Often, the organisation will come in and take the power away from the [family] carer … like, “You’ve done this for however long, but you don’t know what you’re doing. We’re going to come in and do this.” So I think part of that is letting them along the journey as well. So them sharing their experiences* (Katie, service manager).

*Understanding the carer relationship and being able to not be paternalistic, you’ve really got to park your bias* (Janet, case manager)

### Theme 4. Home care workers knowing positive practical strategies to change behaviors

Carers (paid and unpaid) consistently noted home care workers needed to know how to best respond to some of the distress and subsequent behaviour responses people living with dementia may experience as a response to unmet needs, including the behavioural and psychological symptoms of dementia. More understanding of the causes, and positive practical strategies for responding were considered important. Exemplars of strategies, including communication strategies, from the three groups:

*“Don’t ask a question in the first place, just make a statement. It’s time to have a cup of tea, or it’s time to get dressed. Not, “Are you ready to get dressed now? Because they might say no and then what do you do?”* (Erica, family carer)

*“You might take [person with dementia] out and there's a situation that occurs and it's usually a behavioural challenge. So, how are we going to respond? How are we going to calm the person down?”* (Louise, home care worker)

*“[They need] strategies around behaviours … just to understand a little bit more around what’s going on in the brain. So, giving examples of if you get this behaviour, then try this. Or if you get this behaviour, try this. So that they can relate back and say, “I’ve had a client where that happened to me. Next time, I will do that.” So definite strategies stuff … how they can set up an environment. How they can change the way that they react. How they can communicate with families as well as with the care recipient.”* (Katie, service manager).

Not only would a better understanding of behaviour and practical positive response strategies increase home care workers’ confidence it would also help to provide quality person-centred care. Home care workers and managers said they particularly needed strategies for: when a person becomes angry, continually repeats something, and de-escalating challenging situations.

*“I know that people can get quite physically and verbally aggressive, but it would be good to know, ‘Okay, this is the path for that person’. [Home care workers should have] tips and suggestions [such as], “If your client is saying this and repeating the same things over and over, what you might do is this…”* (Grace, home care worker).

*“How to manage certain behaviours, or how to deflect from a behaviour, is a good skill [for good care]. So, if somebody is being repetitive or becoming agitated, how to be able to calmly divert them on to something that's more pleasant, and just break that thread without getting into [an argument] with them”* (Georgia, service manager).

This theme was not highlighted by people living with dementia.

### Theme 5. Effective workplace policies, continuity of care, and workforce culture are important

All participant groups thought effective workplace policies and workforce culture (organizational and overall service model culture) were imperative to home care workers’ provision of good dementia care. Participants repeatedly raised the negative influences of: 1) poor employer/employee communications, 2) strict enforcement of certain workplace policies, and 3) the transient nature of the workforce, which affected continuity of care.

Firstly, poor communication between home care workers and service provider managers hindered quality care. Participants commented about care being adversely affected by home care workers receiving outdated or incomplete client data from the organisation, and very little information provided about what to expect apart from specific tasks to be completed. Funding is linked to task completion, which was often mentioned by all groups as a barrier to good care, resulting in home care workers not spending enough time at each client’s home. Participants stated:

*“[They don’t spend long and rush off to] the next person in the queue, flying in and out in 10 minutes”* (Debra, person living with dementia).

*“There’s a huge lack of communication between all the agencies, and an even bigger lack of communication between the agencies and the workers. And that’s in all the agencies we’ve dealt with”* (Caroline, family carer).

*“[Our work] is very much task focused. So we don’t get information about the client’s mental health or state of dementia. We need much more information about the clients and more up-to-date information. Some clients, I look at their care notes and I think ‘Gee I’m not sure when they were doing that, but they’re a long way from that now!*’” (Grace, home care worker).

Secondly, participants perceived the strict enforcement of certain workplace policies limited home care workers’ capacity to provide quality care. For example, workers were rarely paid for travel time between clients, which imposed time constraints. Both family carers and home carers were frustrated about the strictly enforced policy of not being able to share contact numbers, with communication between them having to be triaged through office-based staff, which often led to missed information/misunderstandings. For example:

*“It just got to the point that I could never identify who was here on what day and therefore who was responsible for whatever went wrong. I think one of the frustrations for the carer is you have absolutely no contact. The agencies are very strict on no mobiles, but it means that [you’ve got no contact.]”* (Suzy, family carer)

*“There are guidelines at work, [stating] “they are not our friends. We don't have their phone numbers, and they can't have ours”. But there is an emotion there that no paper or rule can take away”* (Lily, home care worker)

Thirdly, consistency between home care workers and continuity of care was significantly valued by all participants for good care provision. People with dementia and family carers found changes in rostered workers - which happened frequently and often abruptly with no explanation - frustrating and confusing. Home care workers also found these changes reduced their capacity to provide good care, and they felt frequent changes led to poor consistency, and inability to enact routine and maintain relationships. They also mentioned the difficulty of not knowing what to expect at each client’s home. For example,

*“The home care worker was really fantastic too. Then they just disappeared – they came one day and you never saw them again. They didn’t even say goodbye.”* (Debra, person living with dementia)

*“It's variation all the time … it's totally different to anything I'm used to. The people that come to see me … they’re different. I don’t know why”* (Trevor, person living with dementia).

*“I’ve got a different person coming again today. That’s the one thing, the lack of continuity, that’s very frustrating… it’s three days, three different people. Whenever there is someone new, I’ve taken mornings off work so I can at least meet the person”* (Helen, family carer)

*“[My husband] didn’t need to have different people all the time. It’s very confusing for someone who has no short term memory to have different people coming in all the time. They get very anxious and they’re also very suspicious.”* (Francis, family carer).

*“Not consistent enough, again because each carer of course has their own way of caring and showering … yeah, there’s a lot of inconsistency.”* (Ava, home care worker).

Managers agreed that not being able to regularly assign home care workers to particular clients affected the quality of dementia care provided.

“*The continuity is not there. And for dementia patients that’s what you need. So you need to keep reintroducing these three people coming in and out. It’s not easy. It’s very hard*” (Milly, case manager).

*“A lot of the carers will juggle two or three agencies... trying to jigsaw-puzzle together a roster. Which I totally understand, because if you need to earn money, you need to earn money. In a perfect world, it would be great if every carer just worked for us and we could give them enough work and give them regular updates for things that they needed, and they were more stable. But they're casual.”* (Georgia, Service manager)

All stakeholder groups voiced their frustration at the constraints of an often transient, casualised workforce that was poorly paid, which affected the ability of home care workers to provide both continuity of care and the ability to form long-term relationships with care recipients. For example:

*“The pay is lousy; really lousy. Because I’ve heard sort of ‘I can’t afford this now’…They don’t pay them well and they don’t look after them.”* (Debra, person living with dementia)

“*I realised it’s a very transient industry... I just keep plugging away looking for the right people that will sort of fit in and be longer term”* (Suzy, family carer)

*“It’s a casual workforce with all that brings to it – so consistency is an issue and reliability and finding good care workers is difficult. They’re obviously not paid enough – you know for what they do, it would be great if they could be paid more.”* (Marie, service manager)

## Discussion

This is the first study to explore the perceptions of all stakeholders who receive and provide in-home care services, with the aim of investigating what they perceive constitutes good dementia care by home care workers. We have previously reported on care recipients’ experiences of receiving community care to address the need for specialist dementia training for home care workers [10]. Current findings indicate that care recipients and care providers have highly similar priorities in care. Thematic analysis discovered a high rate of consensus between stakeholder groups, with five clear themes across groups that they thought was important for good care: 1) Home care workers’ understanding of dementia and its impact; 2) Home care workers’ demonstrating person-centred care and empathy; 3) Good relationships and communication between care worker, person with dementia and family carers; 4) Home care workers’ knowing positive practical strategies for changed behaviours; 5) Effective workplace policies and workforce culture. Thematic analysis did not indicate any differing or competing priorities for good care in each group, with the exception of knowledge about changed behaviours, which was not a theme identified in the group living with dementia.

In our study, all stakeholders considered it crucial that home care workers should have a fundamental level of dementia knowledge, particularly about how dementia impacts an individuals lived experience (knowledge which they currently lack, and rather than knowledge about the theoretical underpinnings of dementia). However, it is unclear whether this perceived lack of knowledge results from poor training or from a lack of experience, and whether the knowledge should be gained by training or workplace experience (or both). For example, health care aides in a previous study attributed their dementia knowledge and competence to work experience rather than theoretical training and education [31]. Findings are consistent with the previously identified need for improved dementia knowledge and education in this workforce [32, 33] including in our previous work [16, 34, 35]. Australia’s aged care workforce strategy [36] states the lack of attention to dementia care within aged care training programs is a significant barrier to building a skilled workforce, and the recommendations of the Australian Royal Commission into Aged Care Quality and Safety [17] identified the need to upskill the aged care workforce in specialist dementia care.

While Australian home care is designed to be consumer directed, none of the key stakeholders in our study experienced the person being the centre of care. Despite this, it was key for both those providing and receiving care that the care relationship is person-centred – that is, individualised, communicative, empathic, with friendship-like qualities (at the same time as respecting client-professional boundaries). Our findings provide further consolidation that dementia care should shift towards a person-centred, flexible approach in supporting well-being and completion of tasks. Good communication and rapport are key determinants of health and well-being of older people with dementia, and essential to person-centred care [37, 38]. Importantly too, community-based dementia care workers report higher levels of job satisfaction when they can maintain positive relations with clients living with dementia and their families [39], demonstrating a mutually beneficial outcome to all parties involved.

Although mentioned as a clear need for the paid and family carers, the participants living with dementia did not mention the need for home care workers to know practical strategies to change behavior, which may relate to reduced insight into what others see as symptoms of dementia. What is seen as ‘challenging behaviors’ by carers may be an understandable response by the person living with dementia, and thus not regarded as “symptoms”, rather an appropriate response to their environment or an unmet need.

Participants in this study believed improved work conditions, policies, and processes would support home care workers to provide quality dementia care. They were frustrated with inconsistent staff rostering, lack of continuity of workers over time, and poor work conditions for home care workers (which result in a transient workforce and rushed visits, leading to less than optimal care). This is consistent with previous work which found predictability and familiarity is highly valued by people with dementia and family carers [37, 40], and the importance of continuity of care in enabling a level of familiarity and trust that allows care workers to recognise and respond to individual needs (including our work by Low et al. [34]; While et al. [35]). While, Winbolt and Nay [35] found that service recipients (people living with dementia and their family members), perceived that service limitations and inefficiencies within the providers’ organisational structure contrasted with positive experiences related to service responsiveness and team work. They found interpersonal relationships with community care workers were the most important factor in the way services were provided - friendly, trustworthy staff were valued; unreliable, task orientated and poorly trained staff were unwanted.

Our findings reflect six of the ten underpinning values in the theoretical care model developed by Lord and colleagues [21] to best support people with dementia to live independently at home. The values that were also supported by our participants were 1) compassionate, person-centred care, taking into account important relationships; 2) activities and plans should be tailored to the individual; 3) their needs and goals (and also that of the family carer) should inform care; 4) strategies were important; and people highly valued good communication with a 5) single point of contact and 6) continuity of care. However, in contrast to the Lord et al. model, our participants (neither care providers nor recipients) did not see their role as being caretakers of maintaining continued connections with earlier social networks and roles; or to ensure the home and wider environment be as dementia-friendly as possible. Additionally, our participants did not highlight the need to consider self-management or the autonomy and the safety of the person living with dementia. Rather, our participants focused on more practical aspects, such as dementia literacy, practical strategies for changed behaviours, and how more effective workplace policies and workforce culture could improve the care provided.

### Limitations

Participants living with dementia and family carers in this study were recipients of government-subsidised services, not full-fee ‘private’ paying clients -although it is estimated 70% of home care is funded by the government [36]. We also recruited all participants via partner service providers, so staff more engaged in dementia care may have volunteered to be involved. All home care workers and family carers were women, which does reflect the typical demographic of carers [41] and of the home care work force in Australia (87% of the aged care workforce in Australia are women) [42]. As in other studies, recruitment of people with dementia was difficult and we did not have balanced numbers of participants from different target groups. Although the focus of qualitative research is to provide rich, descriptive data, and generalizability is not a significant concern [43], there were only four people living with dementia that participated in this study, all of whom were diagnosed with Alzheimer’s disease. Further research should focus on gathering data from a wider spectrum of diagnoses and severity of dementia. Future research should also include more diverse participants in general, such as those paying privately for in-home care, and specific culturally diverse populations. Research should also investigate how care recipients and care providers perceive good care in different countries.

## Conclusions

Australian home care workers providing care for people living with dementia need a sound understanding of dementia and its impact in order to practice person-centred care. The relationship between the paid and unpaid members of the care team is critical to good quality care, as are effective workplace policies and workforce culture. These elements of care provision should be central to the design of home care for people living with dementia and to home care worker training and professional development. This study adds to the evidence about what constitutes quality in-home services for those living with dementia, according to multiple stakeholder groups. Findings can inform the development of workforce training, models of care, policy development, service delivery, and guide future research.

## Data Availability

The datasets generated and/or analysed during the current study are not publicly available due to confidentiality reasons and an on-going RCT but are available from the corresponding author on reasonable request.

## Abbreviation

PITCH: Promoting Independence Through quality dementia Care at Home
